# Interactions between arsenic exposure, high-fat diet and NRF2 shape the complex responses in the murine gut microbiome and hepatic metabolism

**DOI:** 10.3389/frmbi.2022.1041188

**Published:** 2022-11-23

**Authors:** Gabriele Schiro, Pengfei Liu, Matthew Dodson, Donna D. Zhang, Fayez K. Ghishan, Albert Barberán, Pawel R. Kiela

**Affiliations:** ^1^ Department of Environmental Science, University of Arizona, Tucson, AZ, United States; ^2^ Department of Pediatrics, University of Arizona, Tucson, AZ, United States; ^3^ Department of Pharmacology and Toxicology, College of Pharmacy, University of Arizona, Tucson, AZ, United States; ^4^ National and Local Joint Engineering Research Center of Biodiagnosis and Biotherapy, and International Joint Research Center on Cell Stress and Disease Diagnosis and Therapy, The Second Affiliated Hospital of Xi’an Jiaotong University, Xi’an, China

**Keywords:** arsenic, diet, liver, multi-omics, microbiota, NFE2L2 gene

## Abstract

Inorganic arsenic (iAs) exposure has been associated to various detrimental effects such as development of metabolic syndrome and type 2 diabetes *via* oxidative stress and induced prolonged activation of the NRF2 transcription factor. Such effects can be aggravated by poor dietary habits. The role of gut microbiota in promoting metabolic changes in response to arsenic has yet to be precisely defined. To address the complexity of the interactions between diet, *NFE2L2*/NRF2, and gut microbiota, we studied the chronic effects of iAs exposure in wild-type (WT) and *Nrf2^-/-^* mice fed normal (ND) vs. high-fat diet (HFD), on the gut microbial community in the context of hepatic metabolism. We demonstrate that all treatments and interactions influenced bacteria and metabolic profiles, with dietary differences causing a strong overlap of responses between the datasets. By identifying five metabolites of known microbial origin and following their fate across treatments, we provide examples on how gut microbial products can participate in the development of iAs and HFD-induced metabolic disease. Overall, our results underline the importance of the microbial community in driving gut-liver-cross talk during iAs and HFD exposure.

## Introduction

Environmental exposure to iAs, especially in drinking water, represents a worldwide health risk. Among the detrimental effects of chronic iAs exposure is a higher risk of developing metabolic syndrome (MS) and type 2 diabetes (T2D). In adult mice, sodium arsenite induced glucose intolerance without changes in body weight (Paul et al., 2007). The same form of chronic iAs exposure in adult mice potentiated the effects of high-fat diet (HFD) on glucose intolerance (Paul et al., 2011). Moreover, chronic exposure to elevated iAs during pregnancy in mice led to increased body weight and adiposity, higher serum cholesterol levels, hyper-leptinemia, and hyper-insulinemia in the offspring extending into adulthood (Ditzel et al., 2016; Rodriguez et al., 2016). Arsenic exposure has been observed to induce prolonged activation of the nuclear factor erythroid 2 p45-related factor 2 (NFE2L2 ***/*** NRF2) (Lau et al., 2013; Dodson and Zhang, 2017), a transcription factor that regulates gene expression through the interaction with the antioxidant response element (ARE). NRF2 is known to control cellular mechanisms of defense against oxidative and inflammatory stresses, and its activation leads to the upregulation of protective enzymes that play a critical role in the resolution of inflammation (Vasileva et al., 2020). While activation of NRF2 by iAs has been generally considered to be an adaptive mechanism to reduce cytotoxicity associated with increased reactive oxygen species (ROS), chronic and uncontrolled NRF2 activation has been postulated to promote cancer progression, metastasis, and resistance to therapy (Lau et al., 2008; Rojo de la Vega et al., 2016). We recently demonstrated that iAs diabetogenicity is associated with a prolonged non-canonical NRF2 activation *via* p62-mediated sequestration of KEAP1 (Liu et al., 2021). This mechanism was responsible for the increased carbohydrate flux through the polyol pathway in the liver and led to a pro-diabetic shift in glucose homeostasis.

Finally, there is a substantial body of literature that points to gut microbiota as the trigger of low-grade “metabolic” inflammation, promotion of metabolic syndrome and T2D (Chassaing and Gewirtz, 2014; Scheithauer et al., 2020; Gurung et al., 2020). Gut microbiota may play a central role in promoting metabolic changes in response to iAs and poor, high-fat, low-fiber diet, dominant in western societies. Indeed, HFD is one of the most potent modifiers of gut microbiota and a driver of chronic diseases in mice and humans (Daniel et al., 2014; Carmody et al., 2015; Murphy et al., 2015). Exposure to environmental pollutants, such as particulate matter and heavy metals, are also known to alter the composition of the gut microbiome, leading to disease (Bailey et al., 2020; Arun et al., 2021). The main route of iAs intake is through the digestive trait (Calatayud and Llopis, 2015). Thus, it is not suprprising that chronic iAs exposure in adult mice, or in mice exposed to iAs *in utero* and early postnatal development, can significantly affect gut microbial composition (Lu et al., 2014; Chi et al., 2017; Chiocchetti et al., 2019; Wu et al., 2022). Conversely, the gut microbiome is known to influence the fate, toxicity and mobility of iAs species and metabolites, ultimately influencing the effects of iAs on the host (Lu et al., 2013; Yang et al., 2021). Gut bacteria also remain in a reciprocal relationship with NRF2. They lead to the activation of hepatic NRF2 (Saeedi et al., 2020), whereas deactivation of the NRF2 pathway in *Nrf2^-/-^* mice alters the microbial composition in the gut, which suggests a complex feedback affecting the regulatory activities performed by the NRF2 pathway and the gut microbiome (Song et al., 2021).

However, our current knowledge about the role of iAs, high-fat diet, or the role of NFE2L2 ***/*** NRF2 largely comes from reductionist approaches with isolated effects, even though their interactions are likely responsible for the final phenotypic outcomes in individual patients or in vulnerable communities. In this study, we attempted the challenging task of integrating the effects of iAs, normal (ND) and high-fat diet (HFD), and NFE2L2 ***/*** NRF2 genetic status on the gut microbial community in the context of hepatic metabolism.

## Methods

### Animal experiments

*Nrf2* ^+/+^ (wild type, WT) and *Nrf2^-/-^* mice were generated by breeding *Nrf2* ^+/-^ mice in the C57BL/6J background and the Littermates were used in the study. Eight-week-old mice (25-27g) were randomly allocated to the Ctrl group, or iAs group, HFD group and HFD+iAs group (n= 5 mice per group). Mice in the Ctrl group received normal drinking water, while mice in the iAs group received drinking water containing sodium arsenite (25 ppm) for 20 weeks. Water with iAs was changed to a fresh solution twice a week to ensure iAs stability. 25 ppm sodium arsenite was chosen as it reflects the amount commonly used in the literature to obtain diabetic phenotypes (Paul et al., 2008). The mice in HFD group were fed with high fat diet (Research Diets, D12492) containing 60% calories from fat, 20% from protein and 20% from carbohydrates for 20 weeks, while the mice in other normal diet groups received the normal chow (Envigo Teklad Laboratory Diet, 2018) containing 18% calories from fat, 24% from protein and 58% from carbohydrates. Fecal samples from all 40 mice were collected one week before mice were sacrificed. All mice were euthanized, and liver tissue was collected at 20 weeks of treatment. Physiological data (body, liver, spleen weight, body fat, blood volume, and glucose level), was registered at the end of the 20-week study.

### Microbiome analyses

Genomic DNA was extracted from 0.25g of fecal matter, using a FastDNA Spin Kit for Soils (MP Biomedicals, OH, USA), following the manufacturer instructions. Extraction blanks were included to control for potential contaminations during the extraction and sequencing procedures. We amplified the V4 hypervariable region of the 16S rRNA gene by PCR using the 515-F (GTGCCAGCMGCCGCGGTAA) and the 806-R (GGACTACHVGGGTWTCTAAT) primer pair (Caporaso et al., 2012). The primers included Illumina adapters and an error correcting 12-bp barcode, that, unique to each sample, was used for demultiplexing. An Ultra-Clean PCR Clean-Up kit was used to clean PCR products (MoBio Laboratories, Carlsbad, CA, USA). Quant-iT PicoGreen dsDNA iAssay Kit was used for product quantification (Invitrogen, Waltham, MA USA). Purified DNA products were pooled together in equimolar concentration and sequenced on a 2x150 bp Illumina MiSeq platform (Illumina, San Diego, CA USA) at the Microbiome Core Steele Children Center, University of Arizona, USA. Demultiplexing was performed using idemp (https://github.com/yhwu/idemp). Demultiplexed files were used to generate amplicon sequence variants (ASVs) with the DADA2 pipeline (Callahan et al., 2016). The reads were trimmed to 140 base pairs and reads exceeding a maximum expected error of 2 or more base pairs were removed. The resulting quality-filtered reads were used to train the error model in DADA2. Paired-end reads were merged, and chimera sequences removed. Taxonomic identities were assigned using the Ribosomal Database Project (RDP) classifier (Wang et al., 2007), on the SILVA nr version 132 database (Quast et al., 2012). Any ASVs that were assigned to chloroplast, mitochondrial, or archaeal origin were removed. We also removed samples with less than 10,000 sequences, leaving a total of 39 samples.

### Metabolomics

Metabolomic analyses were performed as described (Liu et al., 2021). Briefly, liver tissue from 20-week-old mice was collected, frozen in liquid nitrogen, and subsequently shipped to Metabolom (Morrisville, NC, USA), for further analyses. Methanol precipitation and centrifugation was used to remove proteins. The final extract was analyzed using ultrahigh performance liquid chromatography-tandem mass spectroscopy (UPLC-MS/MS). Peak identification, validation, and scaling (median equal to 1) were performed by Metabolon. Data was multiplied by a factor 10000 and then log2 transformed to achieve homoscedasticity. Among the metabolites measured, five were selected to be used as indicators of the activity of the gut-liver axis: (1) 3-indoxyl sulfate is a liver metabolite of indole, a gut bacteria produced metabolite (Zgoda-Pols et al., 2011; Huć et al., 2018). (2) Imidazole propionate is a gut microbial product that, once absorbed through the portal vein into the liver, enters systematic circulation and contributes to type 2 diabetes parthenogenesis (Koh et al., 2018). (3) Trimethylamine N-oxide (TMAO) is derived from the oxidation of trimethylamine (TMA), a bacterial metabolite of choline. TMA is absorbed in the intestine and delivered in the liver, where it is metabolized into TMAO (Arias et al., 2020). (4) Deoxycholate is a secondary bile acid, with putative mediative cancerogenic effects and (5) N,N,N-trimethyl-5-aminovalerate (TMAVA) a microbial product which has been reported to promote liver steatosis in mice consuming a high fat (Zhao et al., 2020).

### Statistical analyses

Most statistical analyses were implemented in R (T.R.D. Core, 2020) using vegan for multivariate statistics (Oksanen et al., 2018). Alpha diversity metrics (ASV richness, Shannon diversity) were calculated on rarefied data (81,000 sequences) to reduce biases due to differential sequence counts. Differences in richness and diversity among treatments were tested using a 3-way ANOVA with interactions included in the models. To evaluate the effects of the treatments on microbial community profiles, Bray-Curtis dissimilarities were calculated on CSS scaled ASV tables. Permutational analysis of variance (PERMANOVA) was used to calculate compositional differences among treatments, including interactions among variables (Anderson, 2001; Paulson et al., 2013). To evaluate the effect of the treatments on mice liver metabolic profiles, Euclidean distances were calculated, and PERMANOVA was used to assess differences among treatments. To identify differentially abundant ASVs, we used the R package DESeq2 (Love et al., 2014), with a P < 0.05 after FDR correction. Variation partitioning analysis was used to evaluate the effect of the treatments in driving the observed correlations (Peres-Neto et al., 2006). In this case, Euclidean distances between metabolic profiles were used as response variables, while the first ten axes of the NMDS ordination were used as representative of the microbial community. Metabolite differential abundance between conditions were tested fitting linear regressions with interactions included in the models (P < 0.05 after FDR correction). Correlations between indicator metabolites and phylotypes (50 most abundant) were calculated using Spearman correlations (P < 0.05 after FDR correction). Additional statistical analyses of non-microbiome data were performed with GraphPad Prism 9 software, as described in figure legends.

## Results

### Phenotypic analyses

PERMANOVA was calculated on Euclidean distances based on the phenotypic characteristics (weight gain from the start till the end of the study, average calorie consumption, and blood glucose, and liver, spleen, kidney, and abdominal fat pad weights data collected at the time of euthanasia). The measured phenotypical characteristics were significantly affected by diet and genotype, while iAs intake did not show any statistically significant effect alone. iAs intake was only significant when interacting with diet (**Supplementary Table S1**). Predictably, HFD led to increased caloric intake and increased baseline glucose levels in each experimental group (**Supplementary Figure S1A**). Body weight gain was consistently elevated in HFD-fed mice compared to mice fed control diet, but the gain in response to HFD was significantly lower in iAs-exposed mice regardless of their genotype (**Supplementary Figure S1A**). HFD diet led to increased liver, spleen, and abdominal fat pad, but not kidney weights (**Supplementary Figure S1B**). In *Nrf2^-/-^* mice, iAs blunted the effects of HFD on liver weight. iAs exposure also significantly blunted the effects of HFD on abdominal fat pad weight regardless of the genotype and a similar effect was observed for the spleen weight, although without reaching a statistical significance (**Supplementary Figure S1B**). Kidney weight was not affected by any of the experimental variables.

### Taxonomic composition and richness of fecal microbiota

**Figure 1:**
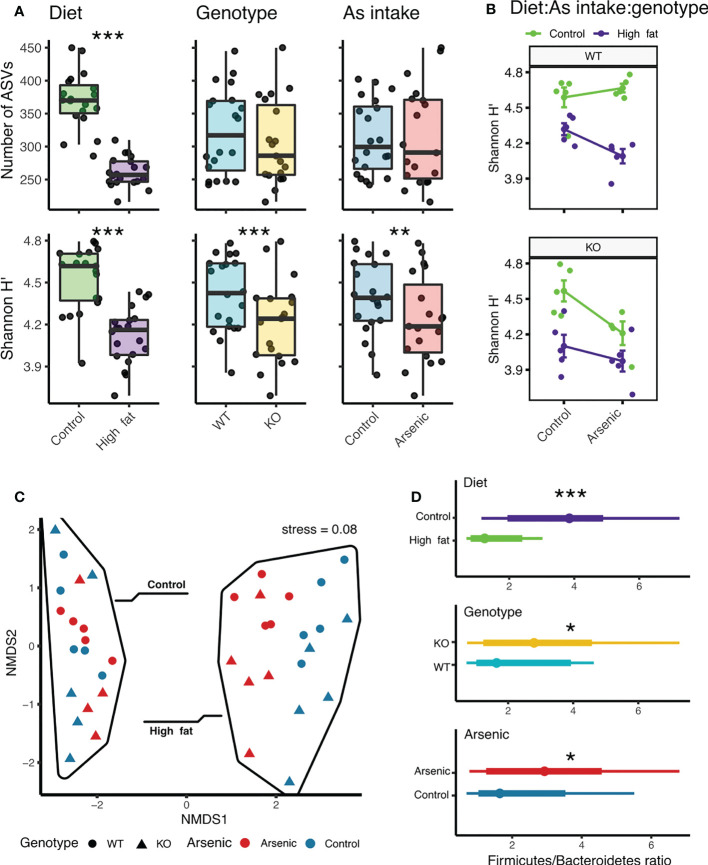
Influence of diet, iAs exposure and NFE2L2*/*NRF2 status on general indices of microbial diversity. **(A)** Boxplots showing differences in richness and Shannon diversity across treatments. **(B)** Shannon H’ is influenced by the interaction between diet, genotype, and arsenic. **(C)** NMDS ordination based on microbiome composition. **(D)** Differences in Firmicutes to Bacteroidetes ratios across treatments (*P < 0.05, **P < 0.01, ***P < 0.001).

The total richness (number of ASVs) was 1,529. The average richness per sample, after rarefaction, was 315 ( ± 65) ASVs. At the phylum level, microbial communities were dominated by Firmicutes (61%), Bacteroidetes (29.9%), Proteobacteria (3.1%), Verrucomicrobia (2.3%) and Tenericutes (1.9%). Diet was the only factor with a significant effect on richness (**Table 1**). For Shannon diversity, statistically significant differences were detected between different diets, iAs exposure, and genotypes (**Table 1**). The interaction between all three factors was also found to be significant (**Table 1**), while the other two interactions were not. All treatments and all interactions showed a significant effect on microbial community compositional similarities, with diet being the strongest predictor, followed by genotype and by the interaction of all three variables (**Table 1**; **Figure 1C**). HFD, but not iAs exposure or NFE2L2 ***/*** NRF2 status significantly reduced microbial diversity expressed as the number of ASVs (**Figure 1A**). However, Shannon’s index, which accounts for both abundance and evenness of the species present, was reduced by each of the three experimental variables (**Figure 1A**). Interestingly, while iAs intake in ND wild-type mice modestly increased alpha diversity (Shannon H’ index), deletion of NRF2 led to a reciprocal effect, thus indicating that NRF2, at least to some extent, protected the gut microbiota from the dysbiotic effects of iAs (**Figure 1B**). All treatments led to a reduced Firmicutes/Bacteroides ratio (**Table 1**), while interactions were non-significant (**Figure 1D**; **Table 1**).

**Table 1:** ANOVA and PERMANOVA results for microbiome and metabolome profiles.

Single variate												
	Richness				Shannon H’				Firmicutes/Bacteroidetes ratio			
Ind. Var.	Df	Sumsq	F	P	Df	Sumsq	F	P	Df	Sumsq	F	P
Genotype	1	2857	2.44	0.13	1	0.39	13.67	**<0.001**	1	8.71	5.14	**O.03**
Diet	1	115781	99.07	**<0.001**	1	1.56	53.71	**<0.001**	1	43.01	25.37	**<0.001**
As	1	129	0.11	0.74	1	0.22	7.731	**0.009**	1	8.47	4.99	**0.03**
Genotype : Diet	1	651	0.56	0.46	1	0.081	0.278	0.6	1	0.08	0.48	0.83
Genotype : As	1	46	46	0.84	1	0.592	2.036	0.16	1	0.47	0.28	0.6
Diet : As intake	1	3672	3.142	0.086	1	0.005	0.165	0.69	1	0.53	0.311	0.58
Genotype : Diet:As int.	1	588	0.503	0.48	1	0.18	6.199	**0.018**	1	0.86	0.5	0.48
Multivariate												
	Microbiome				Liver metabolites							
Ind. Var.	Df	F	R^2^	P	Df	F	R^2^	P				
Genotype	1	24.47	0.15	**<0.001**	1	4.02	0.06	**0.009**				
Diet	1	58.25	0.35	**<0.001**	1	25.9	0.37	**<0.001**				
As	1	11.87	0.07	**<0.001**	1	1.80	0.03	0.1				
Genotype : Diet	1	12.36	0.07	**<0.001**	1	1.93	0.03	0.085				
Genotype : As	1	8.82	0.05	**<0.001**	1	3.06	0.04	**0.022**				
Diet : As intake	1	9.42	0.06	**<0.001**	1	1.61	0.02	0.149				
Genotype : Diet:As int	1	8.76	0.05	**<0.001**	1	1.39	0.02	0.206				

Values in bold are significant (P < 0.05).

### Differential abundance analysis of microbiome data

**Figure 2:**
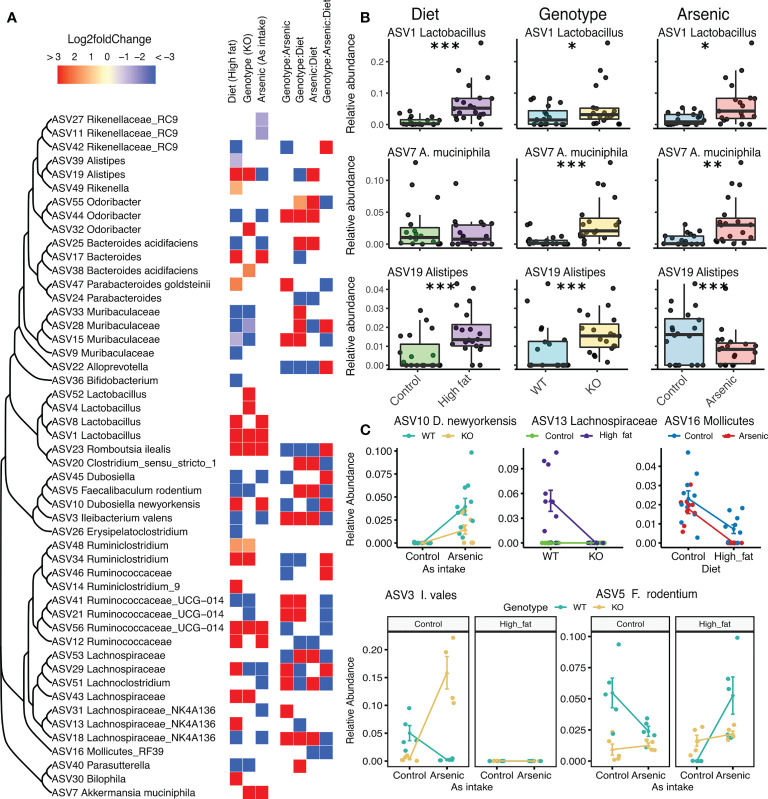
Influence of diet, iAs exposure and NFE2L2*/*NRF2 status on gut microbiota: differential abundance analysis. **(A)** DESeq2 results of the 50 most abundant ASVs found affected by either a treatment or an interaction between treatments. Cells are colored according to the amplitude of the fold change in relationship to the treatment and with the controls as reference, empty cells mean non-significant effects. ASVs are grouped into a tree based on their sequence similarity using a neighbor-joining clustering method. **(B)** Examples of phylotypes being influenced by the three different treatments. **(C)** Examples of phylotypes being influenced by the interaction between treatments. (*P < 0.05, **P < 0.01, ***P < 0.001).

DESeq2 identified that 261 ASVs were changed by diet treatment, 134 by genotype, and 84 by iAs exposure. The interaction between genotype and iAs significantly influenced the abundance of 142 ASVs, between genotype and diet influenced 147 ASVs, between iAs and diet 101 ASVs and all three factors interacting 115 ASVs (**Figure 2A**; a complete list in **Supplementary Tables S2**). Among the most abundant ASVs affected by all three experimental variables was ASV1 Lactobacillus (**Figure 2B**), which showed an increase with all three disturbances. *Akkermansia muciniphila*, a gut bacterium associated with probiotic properties (Cani and de Vos, 2017), increased in *Nrf2^-/-^* mice and in iAs-exposed mice, but not in mice fed with a high fat diet (**Figure 2B**). Other ASVs such as ASV19 *Alistipes*, a genus associated to dysbiosis and inflammation (Parker et al., 2020), was found to increase in high fat diets and *Nrf2^-/-^* mice, but decreased with iAs exposure (**Figure 2B**). Among the ASVs found influenced by the interactive effects (**Figure 2C**), we found members of groups often associated with a variety of host health outcomes.

### Differential abundance analysis of liver metabolites

**Figure 3:**
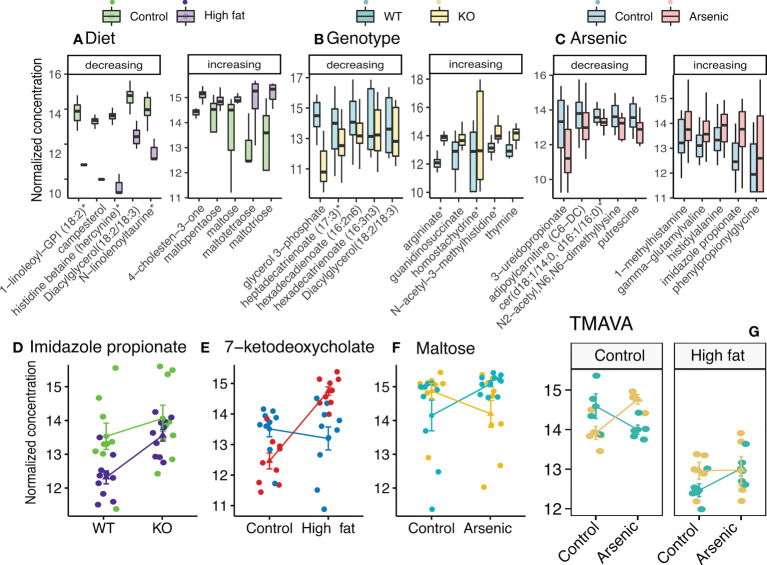
The effects and interaction of diet, iAs exposure, and genotype (NFE2L2*/*NRF2 status) on hepatic metabolites. **(A-C)** Boxplots showing ten (five increasing and five decreasing) most affected metabolites (up- or down-regulated by a respective treatment, as compared to controls). **(D-G)** Examples of metabolites significantly influenced by the interaction between treatments.

A total of 738 metabolites were detected. Linear regressions was then used to identify metabolites which measured concentration varies significantly across treatments. Diet significantly changed 449 metabolites, genotype - 173 metabolites, and iAs - 123 metabolites. Interaction between diet and genotype significantly influenced the concentration of 52 metabolites, between diet and iAs intake - of 61 metabolites, between genotype and iAs - of 105 metabolites. **Figures 3A–C** shows 10 metabolites most strongly influenced by each condition. The interaction between all three factors significantly influenced the concentration of 43 metabolites. Examples of these interactions are presented in **Figures 3D–G** and a complete list of the altered metabolites and their associations with individual or composite experimental variables is reported in **Supplementary Tables S3**. We identified carbohydrates involved in glycogen metabolism (maltose, maltotriose, maltotetraose, maltopentatose) and 4-cholesten-3-one, an oxidation product of cholesterol, associated with high fat diet. In *Nrf2^-/-^* mice, we found a higher accumulation of argininate, which was observed to interfere with anti-oxidant cellular defenses of rats (Delwing-de Lima et al., 2017).

**Figure 4:**
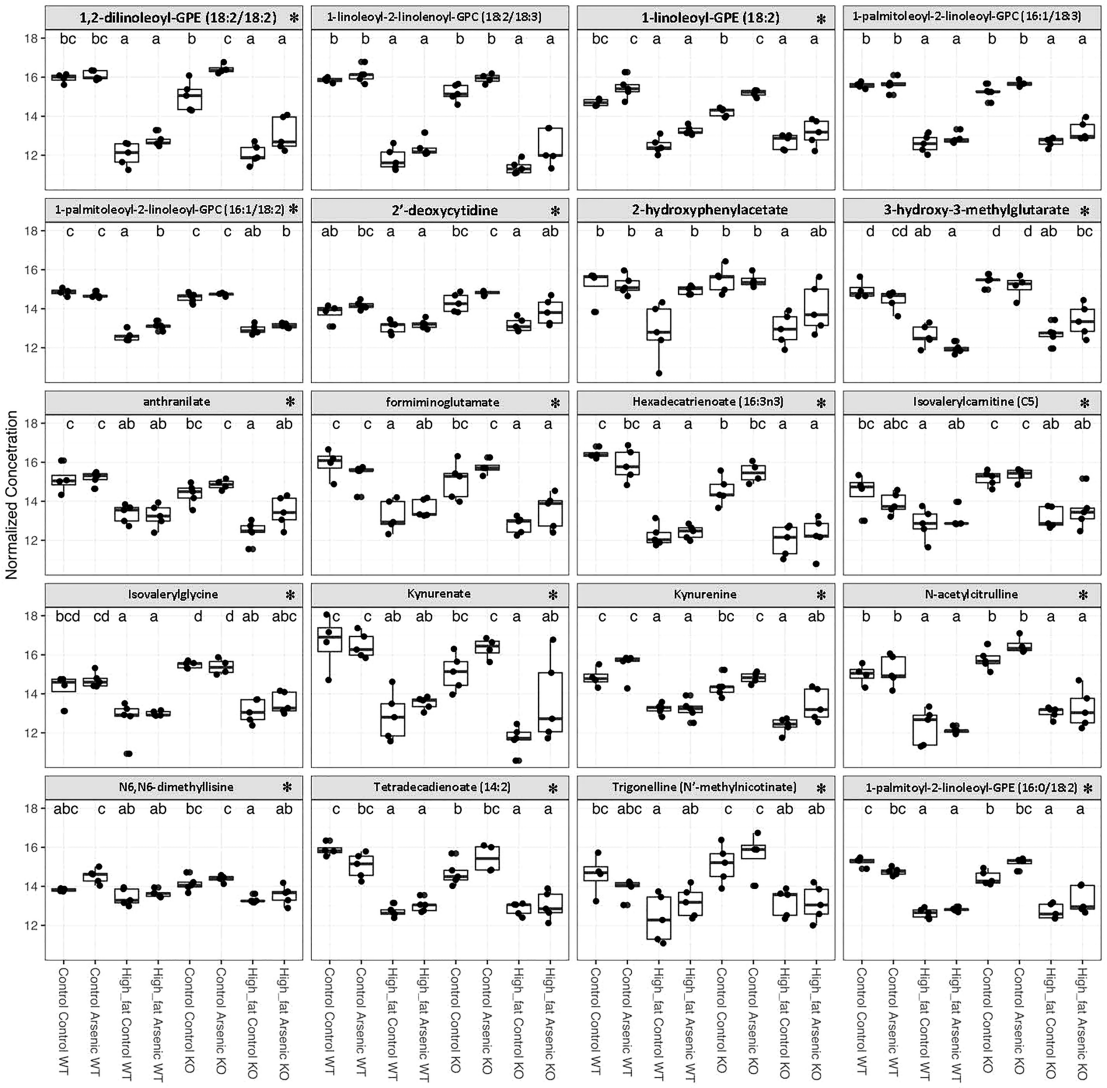
The effects and diet, iAs exposure, and NFE2L2*/*NRF2 status on the most abundant hepatic metabolites. Hepatic metabolites were sorted by their abundance across all samples in the study, and log-normalized values were analyzed by ANOVA. Twenty most abundant metabolites whose concentration was influenced by the treatments (defined by one-way ANOVA; p-adj < 0.05 after FDR correction) were selected for presentation across experimental groups. Within each graph, different letters next to boxes represent statistically significant differences between groups, according to *post-hoc* tests for pairwise significance corrections using Tukey’s HSD contrasts (P < 0.05 as significant). Asterisks next to the metabolite name indicates that it was also identified among fecal metabolites of untreated healthy wild-type mice.

To provide a more two-dimensional glimpse of the hepatic metabolites altered in individual treatment groups, hepatic metabolites were sorted by their abundance across all samples in the study, and log-normalized values were analyzed by ANOVA followed by Tukey’s HSD pairwise comparison. **Figure 4** depicts twenty most abundant metabolites whose abundance was influenced by the treatments in a statistically significant manner. This group is comprised of amino acids, phospholipids, phosphoethanolamines, lipids, a nucleotide, and a phytochemical alkaloid. Interestingly, most of these metabolites (marked by an asterisk in **Figure 4**) were also found by an independent untargeted metabolic screen of fecal samples from treatment-naïve mice (data not shown), thus suggesting that they may have originated from the diet and/or microbial metabolism. With a few exceptions, iAs alone did not affect either of those metabolites regardless of the genotype and the observed changes were driven primarily by diet. The two exceptions were 1,2-dilinoleoyl-GPE (18:2/18:2) and 1-linoleoyl-GPE (18:2) which were significantly induced by iAs only in *Nrf2^-/-^* mice. L-tryptophan (Trp)-kynurenine pathway, also implicated in glycemic control, was represented by kynurenine, kynurenate, and anthralinate. These three likely represent the sum of their production by gut microbiota, and intestinal and hepatic Trp metabolism. The levels of all three were significantly lower in HFD groups (**Figure 4**).

### Relationship between fecal microbiome and liver metabolome

**Figure 5:**
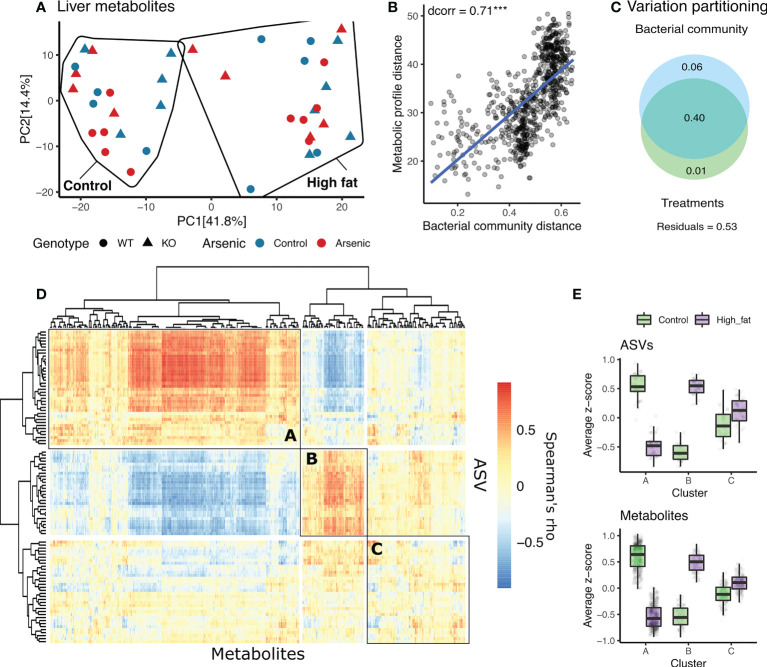
The interaction between gut microbiota and liver metabolites in the context of diet and genotype (NFE2L2*/*NRF2 status). **(A)** PCA based on Euclidean distances between liver metabolites. **(B)** Relationship between Bray-Curtis dissimilarities in microbial communities and Euclidean distances of liver metabolomes. **(C)** Venn diagram representing variation in liver metabolomes in relationship to different treatments (bottom circle) and gut microbial community (upper circle). The percent of variation explained by that set of predictors equals the sum of the numbers in each circle. Numbers in overlaps are equals to the variance jointly explained by them. **(D)** Clustered heatmap of spearman correlations between metabolites (x axis) and the 200 most abundant ASVs across the study (y axis). Within the heatmap three clusters **(A-C)** are formed according to the response of ASVs and metabolites to diet. **(E)** Boxplots of z-scores of ASVs and metabolites clustered in A, B and C, showing a common response to diet. *** indicates P < 0.001.

According to PERMANOVA analysis, diet was the strongest predictor of metabolite composition, followed by genotype and by the interaction between genotype and arsenic intake (**Table 1**, **Figure 5A**). Gut microbiota dissimilarities and liver metabolites distances were highly correlated (**Figure 5B**). Clustering patterns indicate how the correlation between the two matrices was mainly driven by their responses to diet. ASVs and metabolites belonging to cluster “A” generally show a lower abundance in high fat diet and in opposition to those in cluster “B”, while cluster “C” included ASVs and metabolites which did not strongly react to differences in diet (**Figures 5D, E**). Variation partitioning analyses also indicated a strong overlap between the metabolite composition and gut microbial community distances as a response to different treatments (40% co-variation), but further identified 6% of metabolite variation as explained by the gut microbiome independently of the treatments. 1% of the variation induced by treatments did not overlap with differences in the microbial community (**Figure 5C**).

**Figure 6:**
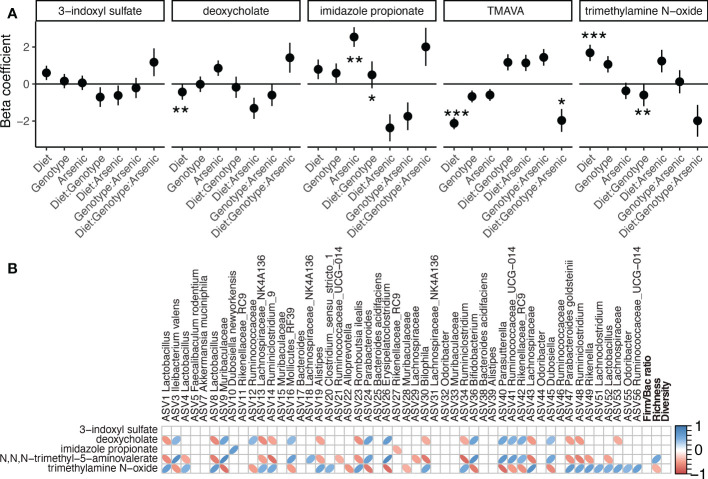
Hepatic microbially derived metabolites in the liver in the context of diet, iAs exposure, and genotype (NFE2L2*/*NRF2 status). **(A)** Responses of selected microbially derived metabolites to different treatments as identified by linear models (*P < 0.05, **P < 0.01, ***P < 0.001 after FDR correction). **(B)** Spearman correlation matrix between the 50 most abundant ASVs and the five selected microbially derived metabolites. Only significant correlations are shown (P < 0.05 after FDR correction).

Besides 3-indoxyl sulfate, all the other selected markers of the microbially mediated gut liver axis activity showed to be significantly affected by either a treatment or an interaction (**Figure 6A**). Imidazole propionate, besides being influenced by iAs, was also influenced by the interaction between diet and genotype. Deoxycholate was reduced in HFD; N,N,N-trimethyl-5-aminovalerate (TMAVA) was also decreased with HFD, while trimethylamine N-oxide (TMAO) was increased. TMAVA was also influenced by all interactions acting simultaneously, while TMAO was also influenced by the interaction between diet and genotype. We then analyzed how these five selected metabolites correlate to members of the gut microbial community (**Figure 6B**). Given that deoxycholate, TMAVA and TMAO responded to different diets, they also correlated with microbial groups that responded to this treatment. For example, deoxycholate and TMAVA both decreased with HFD and showed negative correlations with ASV1-Lactobacillus. TMAVA and TMAO also showed significant and contrasting correlations with richness. 3-indoxyl sulfate did not show correlation to any ASV. Imidazole propionate showed a positive association with ASV10 *D. newyorkensis*.

## Discussion

Arsenic occurs naturally in soil, plants and water and long-term exposure to elevated levels of environmental arsenic has been associated with cancer, heart disease, and other disorders. More recently, it has been suggested that it can act as a metabolic “disruptor” and that the metabolic risks imposed by chronic arsenic exposure, and its ability to promote the development of glucose intolerance and type 2 diabetes, may be underestimated (Kirkley et al., 2018).

### Isolated and combined effects of arsenic exposure, high fat diet and Nrf2 gene knockout on the gut microbial community of mice

All three treatments induced a perturbation in the gut microbial community, causing a reduction of diversity and shifts in community composition. A reduction in microbial diversity is generally associated to a loss of functionality of the community, potentially leading to the development of metabolic diseases (Huttenhower et al., 2012). All three treatments also induced a lower Firmicutes/Bacteroidetes ratio; this has been previously described as a marker of gut dysbiosis in obese patients, although its effective utility has been questioned (Magne et al., 2020). Diet was the treatment inducing the strongest effect on microbial communities, which is in accordance with previous studies point to diet as one of the main determinants of the composition of the gut microbiome in mice and other hosts (Daniel et al., 2014; Singh et al., 2017). Differences in genotype produced a significant variation in the gut microbial community, confirming the results that Nrf2 gene activity can impact gut microbial composition (Song et al., 2021). Arsenic intake had also an effect on microbial composition, as previous studies also observed in mice (Lu et al., 2014; Chi et al., 2017). All tested interactions also showed a significant effect on microbial composition, indicating that the gut community responded differently to the experimental variables when these were acting simultaneously or independently.

The differential abundance analysis showed how all three factors can influence phylotypes with potential implications for host health. ASV1 *Lactobacillus*, the most abundant ASV detected, was observed to significantly increase its abundance with all three disturbances. Strains of *Lactobacillus* are known as possible probiotics, and associated to reductions in body weight in obese patients (Crovesy et al., 2017) but also increase with high fat diets (Daniel et al., 2014). Surprisingly, *A. muciniphila*, showed a higher abundance in KO and iAs- treated mice. *A. muciniphila* has been described as a protective symbiont, which abundance was observed to be inversely correlated to the occurrence of several diseases (Cani and de Vos, 2017; Geerlings et al., 2018). *Alistipes* dysbiosis has been also related to the host’s health, and described as both beneficial and harmful (Parker et al., 2020). Among phylotypes significantly affected by the interactions between treatments, we found members of Lachnospiraceae, which role in relationship to host’s health has been described as controversial (Vacca et al., 2020).

### Correlation of microbial community shifts with hepatic metabolism

We investigated how these changes relate to host liver metabolism, by comparing changes in the gut microbial community to the abundance of hepatic metabolites. We observed that the two matrices show a strong overlap, with diet leading the correlation between the two datasets. This was expected, given that diet is known to strongly affect both gut microbiota and liver metabolism. We showed how microbial metabolites contribute to the differences in the metabolic activity of the host, and that these differences can potentially contribute to the etiology of metabolic diseases. With complex data sets like ours, the reader may best benefit by interrogating our data (in the Supplement or through data repository) for taxa/metabolites of their interest. Due to manuscript limitations, we limited our in-depth analyses to example targets that best reflect the effects of the experimental design. Analysis of the twenty most abundant liver metabolites significantly affected by our experimental variables identified molecules also present in fecal murine samples, suggesting their microbial and/or dietary origin. Although it was evident that high-fat diet was the dominant and often the only modulator of their abundance, two phosphoethanolamines, 1-linoleoyl-GPE (18:2) (LysoPE, HMDB11507) and 1,2-dilinoleoyl-GPE (18:2/18:2) (HMDB0009093), were increased by iAs in *Nrf2^-/-^* but not in WT mice, thus suggesting that Nrf2 modulates liver phosphoethanolamine metabolism in response to chronic iAs exposure. This observation may deserve more scrutiny in the future since there is experimental evidence of an association between phosphatidylethanolamines and insulin sensitivity (Chang et al., 2019). L-tryptophan (Trp)-kynurenine pathway, also implicated in glycemic control, was represented by kynurenine, kynurenate, and anthralinate. These three likely represent the sum of their production by gut microbiota, and intestinal and hepatic Trp metabolism. The levels of kynurenic and anthralinic acids were significantly lower in HFD groups. This correlated with decreased kynurenine, suggesting that the supply of kynurenine, a Trp metabolite, was the primary reason rather than impaired kynurenine metabolism. This observation is in line with the reported contribution of kynurenate (KYNA) to lipid homeostasis. KYNA activates Gpr35 signaling to suppresses weight gain and improve glucose tolerance and adipose tissue inflammation in HFD-fed animals (Agudelo et al., 2018), and decreased KYNA may contribute to impaired glucose tolerance in response to high-fat diet in our experimental setup. However, based on our data, it is unlikely to account for the reported effects of iAs on glucose metabolism (Liu et al., 2021) since iAs or Nrf2 knockout did not modulate the response to HFD.

Multiple linear regression with interactions analysis pointed to additional examples of hepatic metabolites with interactions between iAs exposure, diet, and Nrf2 status. E.g., imidazole propionate, a microbial metabolite of histidine, increased in mice fed with iAs and with the interaction between diet and genotype. Imidazole propionate is known to activate p38γ MAPK signaling pathway, and impair insulin signaling by promoting p62 phosphorylation and activation of mechanistic target of rapamycin complex 1 (mTORC1) (Koh et al., 2018). Imidazole propionate was elevated in patients with prediabetes and diabetes with Bacteroides 2 enterotype, which has previously been associated with obesity, and has been proposed to contribute to type 2 diabetes by modulating host inflammation and metabolism (Molinaro et al., 2020). This may indicate that a similar mechanism may be involved in the etiology of type-2 diabetes *via* associated with poor diet and iAs exposure.

TMAVA and TMAO are microbial metabolites known to contribute to metabolic dysfunction and conditions like liver steatosis, inflammation, and oxidative stress (Arias et al., 2020; Zhao et al., 2020), and were also observed to respond differently to our treatments, although their contrasting behavior is unexpected, since they were both previously associated with liver damage and steatosis (Tripathi et al., 2018; Zhao et al., 2020).

### Summary

All in all, these results show how the gut microbial activity can be perturbated by co-interacting disturbances. All treatments and interactions caused shifts of host gut microbial community, differentially influencing the abundance of bacteria known to affect host’s health. Differences in microbial activity translate into variations of metabolic activity of the liver, underlining the importance of the microbial community in driving gut-liver-cross talk. Our results also suggest how interactions between gut microbes, diet, and arsenic intake can contribute to the development of metabolic diseases. Further in-depth analysis using a wider combination of multi-omics techniques can help develop a better mechanistic understanding of the relationship between the gut microbiome and host metabolism. Such knowledge can contribute to the development of therapeutic approaches for the treatment of various metabolic diseases.

## Data availability statement

Microbiome 16S raw sequence data have been deposited in the NCBI BioProject database under accession # PRJNA875965. Scripts for data analysis can be found at: 364 https://github.com/mrgambero/As_diet_nrf2_mice. Additional raw data can be found in the **Supplementary Material**.

## Ethics statement

The animal study was reviewed and approved by The University of Arizona Institutional Animal Care and Use Committee.

## Author contributions

All authors listed have made a substantial, direct, and intellectual contribution to the work, and approved it for publication.

## Funding

This study was funded by the NIH/NIEHS 5P42ES004940 grant (Raina M. Maier, parent project PI) subproject 5651 (Kiela; Microbial Contributions to Arsenic Transformation in the Gut) and subproject 5649 (Zheng; Diabetogenic Mine Tailings: Mechanistic Link Between Arsenic, NRF2, Autophagy, and Diabetes). The funder was not involved in the study design, collection, analysis or interpretation of the data, the writing of this article, or the decision to submit it for publication.

## Acknowledgments

The authors thank Mrs. Trudy Meckler for her editorial assistance.

## Conflict of interest

The authors declare that the research was conducted in the absence of any commercial or financial relationships that could be construed as a potential conflict of interest.

## Publisher’s note

All claims expressed in this article are solely those of the authors and do not necessarily represent those of their affiliated organizations, or those of the publisher, the editors and the reviewers. Any product that may be evaluated in this article, or claim that may be made by its manufacturer, is not guaranteed or endorsed by the publisher.

## References

[B1] Agudelo L. Z. Ferreira D. M. S. Cervenka I. Bryzgalova G. Dadvar S. Jannig P. R.. (2018). Kynurenic acid and Gpr35 regulate adipose tissue energy homeostasis and inflammation. Cell Metab. 27, 378–392 e5. doi: 10.1016/j.cmet.2018.01.004 29414686

[B2] Anderson M. J. (2001). A new method for non-parametric multivariate analysis of variance. Austral Ecol. 26, 32–46. doi: 10.1046/j.1442-9993.2001.01070.x

[B3] Arias N. Arboleya S. Allison J. Kaliszewska A. Higarza S. G. Gueimonde M.. (2020). The relationship between choline bioavailability from diet, intestinal microbiota composition, and its modulation of human diseases. Nutrients 12, 1–29. doi: 10.3390/nu12082340 PMC7468957 32764281

[B4] Arun K. B. Madhavan A. Sindhu R. Emmanual S. Binod P. Pugazhendhi A.. (2021). Probiotics and gut microbiome – prospects and challenges in remediating heavy metal toxicity. J. Hazardous Materials 420, 126676. doi: 10.1016/j.jhazmat.2021.126676 34329091

[B5] Bailey M. J. Naik N. N. Wild L. E. Patterson W. B. Alderete T. L. (2020). Exposure to air pollutants and the gut microbiota: a potential link between exposure, obesity, and type 2 diabetes. Gut Microbes 11, 1188–1202. doi: 10.1080/19490976.2020.1749754 32347153 PMC7524284

[B6] Calatayud M. Llopis J. M. L. (2015). Arsenic through the gastrointestinal tract. Handb. Arsenic Toxicol., 281–299. doi: 10.1016/B978-0-12-418688-0.00010-1

[B7] Callahan B. J. McMurdie P. J. Rosen M. J. Han A. W. Johnson A. J. A. Holmes S. P. (2016). DADA2: High-resolution sample inference from illumina amplicon data. Nat. Methods 13, 581–583. doi: 10.1038/nmeth.3869 27214047 PMC4927377

[B8] Cani P. D. de Vos W. M. (2017). Next-generation beneficial microbes: The case of akkermansia muciniphila. Front. Microbiol. 8, 1–8. doi: 10.3389/fmicb.2017.01765 29018410 PMC5614963

[B9] Caporaso J. G. Lauber C. L. Walters W. A. Berg-Lyons D. Huntley J. Fierer N.. (2012). Ultra-high-throughput microbial community analysis on the illumina HiSeq and MiSeq platforms. ISME J. 6, 1621–1624. doi: 10.1038/ismej.2012.8 22402401 PMC3400413

[B10] Carmody R. N. Gerber G. K. Luevano J. M. Jr. Gatti D. M. Somes L. Svenson K. L.. (2015). Diet dominates host genotype in shaping the murine gut microbiota. Cell Host Microbe 17, 72–84. doi: 10.1016/j.chom.2014.11.010 25532804 PMC4297240

[B11] Chang W. Hatch G. M. Wang Y. Yu F. Wang M. (2019). The relationship between phospholipids and insulin resistance: From clinical to experimental studies. J. Cell Mol. Med. 23, 702–710. doi: 10.1111/jcmm.13984 30402908 PMC6349352

[B12] Chassaing B. Gewirtz A. T. (2014). Gut microbiota, low-grade inflammation, and metabolic syndrome. Toxicol. Pathol. 42, 49–53. doi: 10.1177/0192623313508481 24285672

[B13] Chi L. Bian X. Gao B. Tu P. Ru H. Lu K. (2017). The effects of an environmentally relevant level of arsenic on the gut microbiome and its functional metagenome. Toxicological Sci. 160, 193–204. doi: 10.1093/toxsci/kfx174 PMC5837326 28973555

[B14] Chiocchetti G. M. Domene A. Kuhl A. A. Zuniga M. Velez D. Devesa V.. (2019). *In vivo* evaluation of the effect of arsenite on the intestinal epithelium and associated microbiota in mice. Arch. Toxicol. 93, 2127–2139. doi: 10.1007/s00204-019-02510-w 31309260

[B15] Crovesy L. Ostrowski M. Ferreira D. M. T. P. Rosado E. L. Soares-Mota M. (2017). Effect of lactobacillus on body weight and body fat in overweight subjects: A systematic review of randomized controlled clinical trials. Int. J. Obes. 41, 1607–1614. doi: 10.1038/ijo.2017.161 28792488

[B16] Daniel H. Gholami A. M. Berry D. Desmarchelier C. Hahne H. Loh G.. (2014). High-fat diet alters gut microbiota physiology in mice. ISME J. 8, 295–308. doi: 10.1038/ismej.2013.155 24030595 PMC3906816

[B17] Delwing-de Lima D. Sasso S. Dalmedico L. Delwing-Dal Magro D. Pereira E. M. Wyse A. T. S. (2017). Argininic acid alters markers of cellular oxidative damage *in vitro*: Protective role of antioxidants. Exp. Toxicologic Pathol. 69, 605–611. doi: 10.1016/j.etp.2017.05.007 28554820

[B18] Ditzel E. J. Nguyen T. Parker P. Camenisch T. D. (2016). Effects of arsenite exposure during fetal development on energy metabolism and susceptibility to diet-induced fatty liver disease in Male mice. Environ. Health Perspect. 124, 201–209. doi: 10.1289/ehp.1409501 26151952 PMC4749082

[B19] Dodson M. Zhang D. D. (2017). Non-canonical activation of NRF2: New insights and its relevance to disease. Curr. Pathobiology Rep. 5, 171–176. doi: 10.1007/s40139-017-0131-0 PMC5654572 29082113

[B20] Geerlings S. Y. Kostopoulos I. de Vos W. M. Belzer C. (2018). Akkermansia muciniphila in the human gastrointestinal tract: When, where, and how? Microorganisms 6, 1–26. doi: 10.3390/microorganisms6030075 PMC6163243 30041463

[B21] Gurung M. Li Z. You H. Rodrigues R. Jump D. B. Morgun A.. (2020). Role of gut microbiota in type 2 diabetes pathophysiology. EBioMedicine 51, 102590. doi: 10.1016/j.ebiom.2019.11.051 31901868 PMC6948163

[B22] Huć T. Nowinski A. Drapala A. Konopelski P. Ufnal M. (2018). Indole and indoxyl sulfate, gut bacteria metabolites of tryptophan, change arterial blood pressure *via* peripheral and central mechanisms in rats. Pharmacol. Res. 130, 172–179. doi: 10.1016/j.phrs.2017.12.025 29287686

[B23] Huttenhower C. Gevers D. Knight R. Abubucker S. Badger J. H. Chinwalla A. T.. (2012). Structure, function and diversity of the healthy human microbiome. Nature 486, 207–214. doi: 10.1038/nature11234 22699609 PMC3564958

[B24] Kirkley A. G. Carmean C. M. Ruiz D. Ye H. Regnier S. M. Poudel A.. (2018). Arsenic exposure induces glucose intolerance and alters global energy metabolism. Am. J. Physiol. Regul. Integr. Comp. Physiol. 314, R294–R303. doi: 10.1152/ajpregu.00522.2016 29118024 PMC5867677

[B25] Koh A. Molinaro A. Ståhlman M. Khan M. T. Schmidt C. Mannerås-Holm L.. (2018). Microbially produced imidazole propionate impairs insulin signaling through mTORC1. Cell 175, 947–961.e17. doi: 10.1016/j.cell.2018.09.055 30401435

[B26] Lau A. Villeneuve N. F. Sun Z. Wong P. K. Zhang D. D. (2008). Dual roles of Nrf2 in cancer. Pharmacol. Res. 58, 262–270. doi: 10.1016/j.phrs.2008.09.003 18838122 PMC2652397

[B27] Lau A. Zheng Y. Tao S. Wang H. Whitman S. A. White E.. (2013). Arsenic inhibits autophagic flux, activating the Nrf2-Keap1 pathway in a p62-dependent manner. Mol. Cell Biol. 33, 2436–2446. doi: 10.1128/MCB.01748-12 23589329 PMC3700105

[B28] Liu P. Dodson M. Li H. Schmidlin C. J. Shakya A. Wei Y.. (2021). Non-canonical NRF2 activation promotes a pro-diabetic shift in hepatic glucose metabolism. Mol. Metab. 51, 101243. doi: 10.1016/j.molmet.2021.101243 33933676 PMC8164084

[B29] Love M. I. Huber W. Anders S. (2014). Moderated estimation of fold change and dispersion for RNA-seq data with DESeq2. Genome Biol. 15, 550. doi: 10.1186/s13059-014-0550-8 25516281 PMC4302049

[B30] Lu K. Abo R. P. Schlieper K. A. Graffam M. E. Levine S. Wishnok J. S.. (2014). Arsenic exposure perturbs the gut microbiome and its metabolic profile in mice: An integrated metagenomics and metabolomics analysis. Environ. Health Perspect. 122, 284–291. doi: 10.1289/ehp.1307429 24413286 PMC3948040

[B31] Lu K. Cable P. H. Abo R. P. Ru H. Graffam M. E. Schlieper K. A.. (2013). Gut microbiome perturbations induced by bacterial infection affect arsenic biotransformation. Chem. Res. Toxicol. 26, 1893–1903. doi: 10.1021/tx4002868 24134150 PMC3974266

[B32] Magne F. Gotteland M. Gauthier L. Zazueta A. Pesoa S. Navarrete P.. (2020). The firmicutes/bacteroidetes ratio: A relevant marker of gut dysbiosis in obese patients? Nutrients. (2020) 12(5):1474. doi: 10.3390/nu12051474 PMC7285218 32438689

[B33] Molinaro A. Bel Lassen P. Henricsson M. Wu H. Adriouch S. Belda E.. (2020). Imidazole propionate is increased in diabetes and associated with dietary patterns and altered microbial ecology. Nat. Commun. (2020) 11(1):5881. doi: 10.1038/s41467-020-19589-w PMC7676231 33208748

[B34] Murphy E. A. Velazquez K. T. Herbert K. M. (2015). Influence of high-fat diet on gut microbiota: a driving force for chronic disease risk. Curr. Opin. Clin. Nutr. Metab. Care 18, 515–520. doi: 10.1097/MCO.0000000000000209 26154278 PMC4578152

[B35] Oksanen J. Blanchet F. G. Friendly M. Kindt R. Legendre P. McGlinn D.. (2020). Vegan: Community ecology package. https://CRAN.R-project.org/package=vegan

[B36] Parker B. J. Wearsch P. A. Veloo A. C. M. Rodriguez-Palacios A. (2020). The genus alistipes: Gut bacteria with emerging implications to inflammation, cancer, and mental health. Front. Immunol. 11, 1–15. doi: 10.3389/fimmu.2020.00906 32582143 PMC7296073

[B37] Paul D. S. Devesa V. Hernandez-Zavala A. Adair B. M. Walton F. S. Drobna Z.. (2008). Environmental arsenic as a disruptor of insulin signaling. Met Ions Biol. Med. 10, 1–7. 20467584 PMC2868343

[B38] Paul D. S. Hernandez-Zavala A. Walton F. S. Adair B. M. Dedina J. Matousek T.. (2007). Examination of the effects of arsenic on glucose homeostasis in cell culture and animal studies: development of a mouse model for arsenic-induced diabetes. Toxicol. Appl. Pharmacol. 222, 305–314. doi: 10.1016/j.taap.2007.01.010 17336358 PMC2680915

[B39] Paulson J. N. Stine O. C. Bravo H. C. Pop M. (2013). Differential abundance analysis for microbial marker-gene surveys. Nat. Methods 10, 1200–1202. doi: 10.1038/nmeth.2658 24076764 PMC4010126

[B40] Paul D. S. Walton F. S. Saunders R. J. Styblo M. (2011). Characterization of the impaired glucose homeostasis produced in C57BL/6 mice by chronic exposure to arsenic and high-fat diet. Environ. Health Perspect. 119, 1104–1109. doi: 10.1289/ehp.1003324 21592922 PMC3237360

[B41] Peres-Neto P. R. Legendre P. Dray S. Borcard D. (2006). Variation partitioning of species data matrices: Estimation and comparison of fractions. Ecology 87, 2614–2625. doi: 10.1890/0012-9658(2006)87[2614:VPOSDM]2.0.CO;2 17089669

[B42] Quast C. Pruesse E. Yilmaz P. Gerken J. Schweer T. Yarza P.. (2012). The SILVA ribosomal RNA gene database project: improved data processing and web-based tools. Nucleic Acids Res. 41, 590–596. doi: 10.1093/nar/gks1219 PMC3531112 23193283

[B43] Rodriguez K. F. Ungewitter E. K. Crespo-Mejias Y. Liu C. Nicol B. Kissling G. E.. (2016). Effects of in utero exposure to arsenic during the second half of gestation on reproductive end points and metabolic parameters in female CD-1 mice. Environ. Health Perspect. 124, 336–343. doi: 10.1289/ehp.1509703 26295903 PMC4786990

[B44] Rojo de la Vega M. Dodson M. Chapman E. Zhang D. D. (2016). NRF2-targeted therapeutics: New targets and modes of NRF2 regulation. Curr. Opin. Toxicol. 1, 62–70. doi: 10.1016/j.cotox.2016.10.005 29082352 PMC5654570

[B45] R Core Team (2020).R: A language and environment for statistical computing. Vienna, Austria: R Foundation for Statistical Computing https://www.r-project.org/

[B46] Saeedi B. J. Liu K. H. Owens J. A. Hunter-Chang S. Camacho M. C. Eboka R. U.. (2020). Gut-resident lactobacilli activate hepatic Nrf2 and protect against oxidative liver injury. Cell Metab. 31, 956–968.e5. doi: 10.1016/j.cmet.2020.03.006 32213347 PMC7329068

[B47] Scheithauer T. P. M. Rampanelli E. Nieuwdorp M. Vallance B. A. Verchere C. B. van Raalte D. H.. (2020). Gut microbiota as a trigger for metabolic inflammation in obesity and type 2 diabetes. Front. Immunol. 11, 571731. doi: 10.3389/fimmu.2020.571731 33178196 PMC7596417

[B48] Singh R. K. Chang H. W. Yan D. Lee K. M. Ucmak D. Wong K.. (2017). Influence of diet on the gut microbiome and implications for human health. J. Trans. Med. 15, 1–17. doi: 10.1186/s12967-017-1175-y PMC5385025 28388917

[B49] Song C. H. Kim N. Nam R. H. Choi S. I. Yu J. E. Nho H.. (2021). Changes in microbial community composition related to sex and colon cancer by Nrf2 knockout. Front. Cell. Infection Microbiol. 11, 1–21. doi: 10.3389/fcimb.2021.636808 PMC8261249 34249773

[B50] Tripathi A. Debelius J. Brenner D. A. Karin M. Loomba R. Schnabl B.. (2018). The gut-liver axis and the intersection with the microbiome. Nat. Rev. Gastroenterol. Hepatol. 15, 397–411. doi: 10.1038/s41575-018-0011-z 29748586 PMC6319369

[B51] Vacca M. Celano G. Calabrese F. M. Portincasa P. Gobbetti M. De Angelis M. (2020). The controversial role of human gut lachnospiraceae. Microorganisms 8, 1–25. doi: 10.3390/microorganisms8040573 PMC7232163 32326636

[B52] Vasileva L. V. Savova M. S. Amirova K. M. Dinkova-Kostova A. T. Georgiev M. I. (2020). Obesity and NRF2-mediated cytoprotection: Where is the missing link? Pharmacol. Res. 156, 104760. doi: 10.1016/j.phrs.2020.104760 32205234

[B53] Wang Q. Garrity G. M. Tiedje J. M. Cole J. R. (2007). Naiüve Bayesian classifier for rapid assignment of rRNA sequences into the new bacterial taxonomy. Appl. Environ. Microbiol. 73, 5261–5267. doi: 10.1128/AEM.00062-07 17586664 PMC1950982

[B54] Wu H. Wu R. Chen X. Geng H. Hu Y. Gao L.. (2022). Developmental arsenic exposure induces dysbiosis of gut microbiota and disruption of plasma metabolites in mice. Toxicol. Appl. Pharmacol. 450, 116174. doi: 10.1016/j.taap.2022.116174 35878798

[B55] Yang Y. Chi L. Lai Y. Hsiao Y. C. Ru H. Lu K. (2021). The gut microbiome and arsenic-induced disease-iAs metabolism in mice. Curr. Environ. Health Rep. 8, 89–97. doi: 10.1007/s40572-021-00305-9 33852125 PMC8728881

[B56] Zgoda-Pols J. R. Chowdhury S. Wirth M. Milburn M. V. Alexander D. C. Alton K. B. (2011). Metabolomics analysis reveals elevation of 3-indoxyl sulfate in plasma and brain during chemically-induced acute kidney injury in mice: Investigation of nicotinic acid receptor agonists. Toxicol. Appl. Pharmacol. 255, 48–56. doi: 10.1016/j.taap.2011.05.015 21640743

[B57] Zhao M. Zhao L. Xiong X. He Y. Huang W. Liu Z.. (2020). TMAVA, a metabolite of intestinal microbes, is increased in plasma from patients with liver steatosis, inhibits γ-butyrobetaine hydroxylase, and exacerbates fatty liver in mice. Gastroenterology 158, 2266–2281.e27. doi: 10.1053/j.gastro.2020.02.033 32105727

