# Supplementary material for: Interactions between arsenic exposure, high-fat diet and NRF2 shape the complex responses in the murine gut microbiome and hepatic metabolism
Source: Front Microbiomes. 2022 Nov 23;1:1041188. doi: 10.3389/frmbi.2022.1041188 (PMC10540274; doi:10.3389/frmbi.2022.1041188)
Supplement: Supplementary file 1 [file DataSheet_1.zip › Supplementary Table S1 and Figure S1AB.PDF]

Supplementary Table S1. Phenotypical similarity patterns. Results of PERMANOVA analyses calculated on Euclidean distances based of mice phenotypical data (weight gain, blood glucose, food consumption, and liver, spleen, kidney, and abdominal fat pad weights). The measured phenotypical characteristics were significantly affected by diet and genotype, while As intake did not show any statistically significant effect alone. As intake was only significant when interacting with diet.

	Phenotype			
**Ind. Var.**	Df	F	R^2	P
**Genotype**	1	7.95	0.06	0.003
**Diet**	1	91.33	0.66	<0.001
**As**	1	2.47	0.02	0.11
**Genotype:Diet**	1	0.66	0	0.5
**Genotype:As**	1	0.4	0	0.7
**Diet:As intake**	1	3.56	0.03	0.04
**Genotype:Diet:As int.**	1	0.05	0	0.99

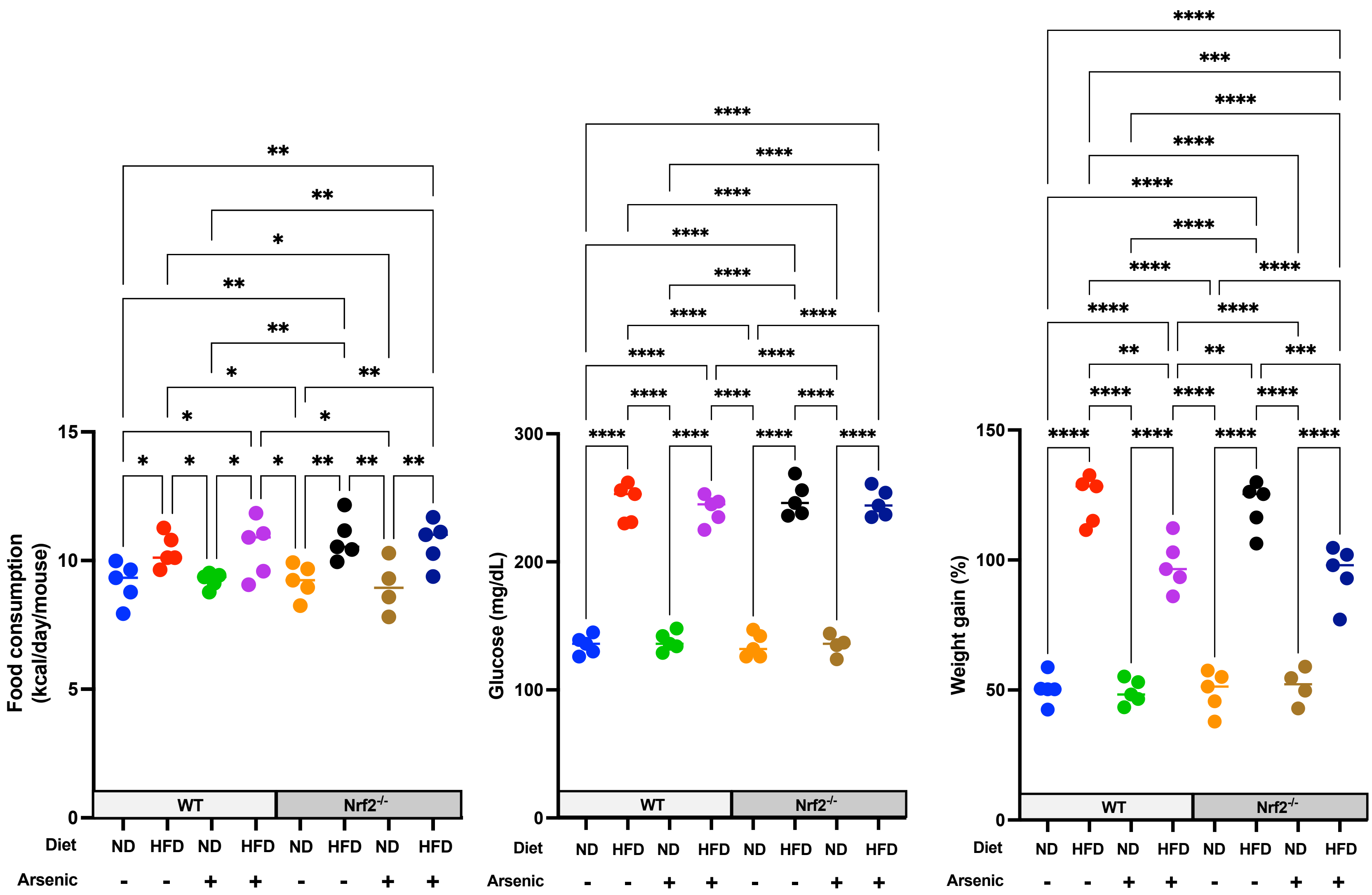

Supplementary Fig. S1A. Daily food consumption during the study (ANOVA=0.0014), blood glucose at the end of the study (ANOVA P<0.0001), and weight gain over the 20 weeks of the study (ANOVA P<0.0001). Asterisks indicate the results of a pairwise comparison with Tukey test with correction for multiple comparisons. ** $P_{adj}<0.005$, *** $P_{adj}<0.001$, **** $P_{adj}<0.0001$. WT – wild-type mice; Nrf2$^{-/-}$ - NFE2L2/NRF2-deficient mice; ND – normal diet; HFD – high-fat diet.

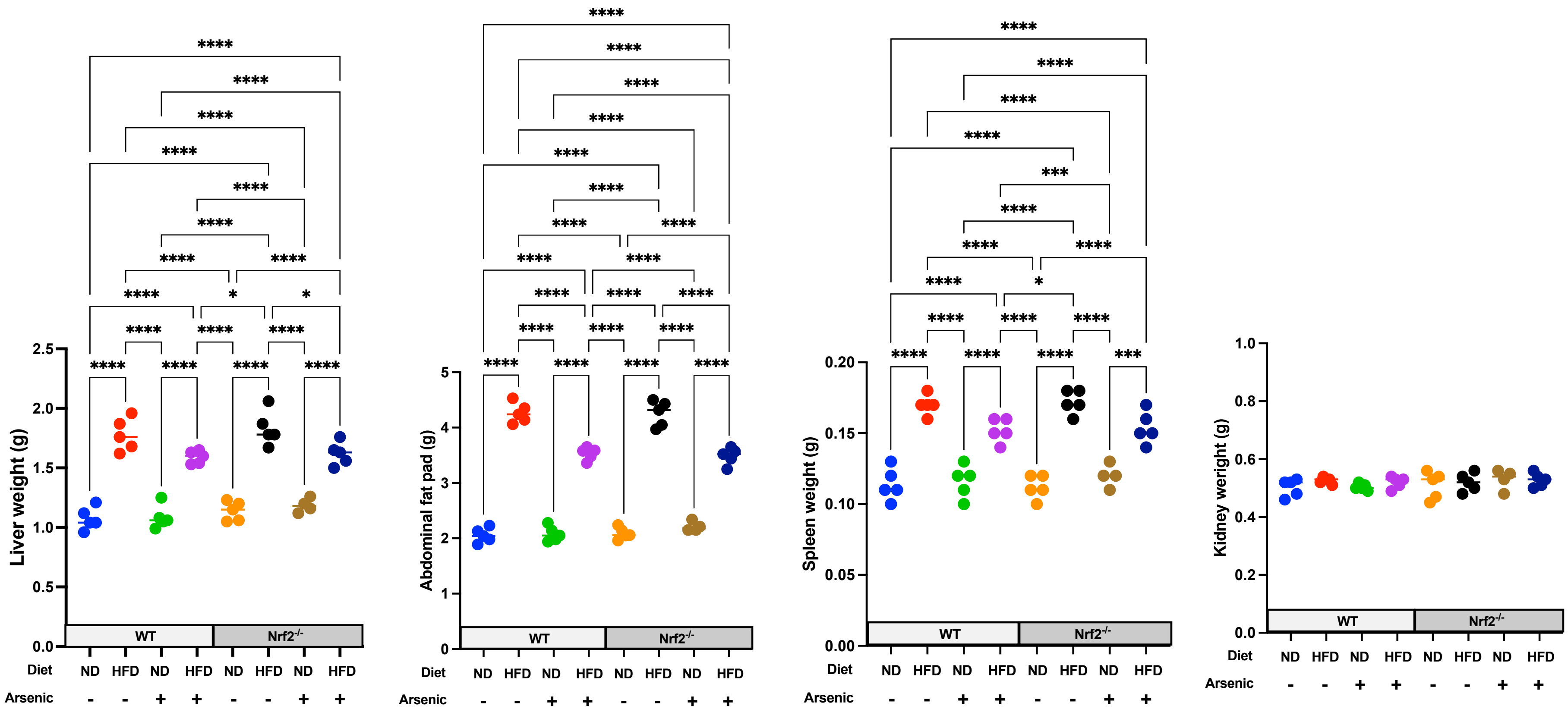

Supplementary Fig. S1B. Liver (ANOVA P<0.0001) , abdominal fat pad (ANOVA P<0.0001) , spleen (ANOVA P<0.0001) , and kidney weight (ANOVA P<0.67) in grams (g) measured at the end of 20-week study. Asterisks indicate the results of a pairwise comparison with Tukey test with correction for multiple comparisons. * $P_{adj}<0.05$, *** $P_{adj}<0.001$, **** $P_{adj}<0.0001$. WT – wild-type mice; Nrf2$^{-/-}$ - NFE2L2/NRF2-deficient mice; ND – normal diet; HFD – high-fat diet.
